# Supplementary material for: Complexome profiling of the Chlamydomonas psb28 mutant reveals TEF5 as an early PSII assembly factor
Source: Plant Cell. 2025 Mar 17;37(6):koaf055. doi: 10.1093/plcell/koaf055 (PMC12164590; doi:10.1093/plcell/koaf055)
Supplement: koaf055_Supplementary_Data [file koaf055_supplementary_data.zip › psb28_interactive.zip!/plots/intensity/qualityPlots/peptides/VTE6_Cre14.g624350.t1.2.html]

### Cre14.g624350.t1.2

Trivial name: VTE6  
SeqCoverage [median]: 16.50  
Mass: 46.83 kDa  
  
 

| Sequence | MissedCleavage | Mass [Da] | wtIntensity | muIntensity | Score |
| --- | --- | --- | --- | --- | --- |
| DANVTGITLSPK | 0 | 1214.65 | 0.00 | 3587.55 | 128.01 |
| EQGVGNVK | 0 | 829.43 | 552.27 | 2585.77 | 120.62 |
| FDFVDEMLR | 0 | 1170.54 | 0.00 | 3702.92 | 82.45 |
| FRDANVTGITLSPK | 1 | 1517.82 | 354.69 | 2486.39 | 47.77 |
| GLMEYGMMTATK | 0 | 1331.59 | 0.00 | 943.01 | 83.87 |
| GTELAKEQGVGNVK | 1 | 1428.76 | 1004.16 | 0.00 | 65.56 |
| KLAPASESAANTQDRVPVGAK | 2 | 2109.12 | 0.00 | 677.67 | 37.29 |
| LAPASESAANTQDRVPVGAK | 1 | 1981.02 | 0.00 | 4910.47 | 73.98 |
|  |  |  | 1911.1 | 18893.8 |  |
